# Stakeholders’ experiences and perception on transitional care initiatives within an integrated care project in Belgium: a qualitative interview study

**DOI:** 10.1186/s12877-023-03746-z

**Published:** 2023-01-23

**Authors:** Merel Leithaus, Amal Fakha, Johan Flamaing, Hilde Verbeek, Mieke Deschodt, Gijs van Pottelbergh, Geert Goderis

**Affiliations:** 1 Academic Center for Nursing and Midwifery, Department of Public Health & Primary Care, KU Leuven, Leuven, Belgium; 2 Department of Health Services Research, CAPHRI Care and Public Health Research Institute, Maastricht University, Maastricht, the Netherlands; 3 Living Lab in Ageing and Long-Term Care, Maastricht, the Netherlands; 4 Division of Gerontology and Geriatrics, Department of Public Health & Primary Care, KU Leuven, Leuven, Belgium; 5 Department of Geriatric Medicine, University Hospitals Leuven, Leuven, Belgium; 6 Competence Center of Nursing, University Hospitals Leuven, Leuven, Belgium; 7 Academic Center for General Practice, Department of Public Health & Primary Care, KU Leuven, Leuven, Belgium

**Keywords:** Integrated care, Interdisciplinary communication, Continuity of patient care, Health care policy, Qualitative research

## Abstract

**Background:**

In 2015, a plan for integrated care was launched by the Belgium government that resulted in the implementation of 12 integrated care pilot project across Belgium. The pilot project Zorgzaam Leuven consists of a multidisciplinary local consortium aiming to bring lasting change towards integrated care for the region of Leuven. This study aims to explore experiences and perceptions of stakeholders involved in four transitional care actions that are part of Zorgzaam Leuven.

**Methods:**

This qualitative case study is part of the European TRANS-SENIOR project. Four actions with a focus on improving transitional care were selected and stakeholders involved in those actions were identified using the snow-ball method. Fourteen semi-structured interviews were conducted and inductive thematic analysis was performed.

**Results:**

Professionals appreciated to be involved in the decision making early onwards either by proposing own initiatives or by providing their input in shaping actions. Improved team spirit and community feeling with other health care professionals (HCPs) was reported to reduce communication barriers and was perceived to benefit both patients and professionals. The actions provided supportive tools and various learning opportunities that participants acknowledged. Technical shortcomings (e.g. lack of integrated patient records) and financial and political support were identified as key challenges impeding the sustainable implementation of the transitional care actions.

**Conclusion:**

The pilot project Zorgzaam Leuven created conditions that triggered work motivation for HCPs. It supported the development of multidisciplinary care partnerships at the local level that allowed early involvement and increased collaboration, which is crucial to successfully improve transitional care for vulnerable patients.

**Supplementary Information:**

The online version contains supplementary material available at 10.1186/s12877-023-03746-z.

## Background

The ageing of the population puts a burden on current health care systems. Older patients with multiple chronic conditions often have complex health care needs over a long period of time. The need for complex health care services frequently leads to health care transitions between different locations or at different levels of care within the same location [1]. Poorly performed healthcare transitions can cause harm, such as poor clinical outcomes, missed diagnosis, incorrect treatment, dissatisfaction among patients, inappropriate use of healthcare services, rehospitalization and mortality [2].

Integrated care initiatives are recommended to ensure continuity of health care and thus particularly benefit older chronically ill patients who often experience transitions. Integrated care is defined as “the management and delivery of health services so that clients receive a continuum of preventive and curative services, according to their needs over time and across different levels of the health system” [3]. Research shows that the integration of health care can result into improved access to services, reduced hospitalizations and readmissions, enhanced adherence to treatment, increased patient satisfaction, improved health literacy and self-care, greater job satisfaction of health care workers, and overall improved health outcomes [4].

Integrated care initiatives that support continuity in care across boundaries require interdisciplinary collaboration, however, establishing consistent multidisciplinary work structures is a complex task [5, 6]. It is known that implementing integrated care programs is difficult as it is often co-determined by unique dynamics and characteristics that can hinder or facilitate implementation within and across health care settings [5, 7]. Studies suggest that attitudes of health-care professionals towards change is crucial. Involving professionals during the development of integrated care programs encourages closer team work early onwards and allows professionals to understand their role as part of the whole [8].

The project ‘Integrated Care for Better Health’ (Integreo) that was launched by the Belgian government in 2016, follows this perspective of including stakeholders in the design of integrated care initiatives [9]. In Integreo, 12 pilot projects for integrated care were designed in co-creation with interested professionals from different local regions [10]. As a result, multidisciplinary local consortia were created to develop a plan with common visions and objectives within a certain geographical area in Belgium. This approach allowed professionals to share their hands on experience while creating a common plan for integrated care. While it is desirable to involve professionals in the development process, it is an extensive process that often leads to challenges, such as additional workload, conflicting opinions and regular changes in scope. These challenges often create an uncomfortable climate of uncertainty, which might create additional burden for health care professionals [10].

This paper focuses on Zorgzaam Leuven (ZZL), one of the 12 Integreo projects [11], by presenting four actions of ZZL that aim to achieve integrated care across settings, thereby improving transitional care and the quality of care interactions. The aim of the paper is twofold. First, we describe the four selected actions that focus on improving transitional care for older chronically ill patients. Second, we aim to get a deeper understanding on the experiences and perceptions of involved stakeholders (health care professionals (HCP) and project coordinators) on transitional care at local level and in the everyday practice.

## Methods

### Design

The study used a qualitative naturalistic case study design, which allows to gain understanding of a complex issue in its real life context [12]. The pilot project ZZL was defined as the case, while the four transitional care actions were selected to study the case. To understand individual perceptions, semi-structured interviews were used as data collection method.

### Study setting

In 2015 the joint plan for chronic care ‘Integreo’ was agreed on by all health ministers in Belgium. This resulted in the start of implementing integrated care pilot projects with the aim to develop and test integrated care initiatives in 12 different regions in Belgium [13]. Each pilot project covers a different geographical region between 75,000 and 360,000 inhabitants. The Belgium government defined 14 components of integrated care to be implemented by the projects. The local project team were allowed to define care goals and the target population in order to implement integrated care where most needed and suited for their region [14]. Thus, new care initiatives can be developed and tested within the 12 projects. The ultimate goal of Integreo is to improve the quadruple aim objectives for healthcare: improving population health and patient experience, reducing costs, and enhancing well-being of health care providers [14, 15].

ZZL is conceptualized as a project to bring lasting change towards integrated care for the region of Leuven, Belgium. The pilot phase of the project took place from 2017 to 2022. ZZL consists of a multidisciplinary local consortium with a core team of ten part-time project coordinators and more than 60 local organizations from the region, including home care organizations, pharmacies, GP practices, regional hospitals, non-profit organizations and research organizations [16]. It covers the region of Leuven and consists of the following sub municipalities: Leuven center, Heverlee, Kessel-Lo, Wijgmaal and Wilsele, including 100,516 inhabitants in October 2021 [17]. Leuven is known as a university city and is therefore characterized by a relatively young population [18]. The percentage of people aged 65+ in Leuven is 16.7% in 2021 [17] while the regional average in Flanders was 20.2% in 2019 [19]. Two hospitals are located in the project region: the regional hospital Heilig Hart Leuven (287 beds) and the University Hospitals Leuven (1995 beds). Within this region ZZL acts as a vehicle to bring different actors together and to promote and initiate change in co-creation.

The multidisciplinary local consortium of ZZL started by conceptualizing a large project plan for the region that was based on three pillars: 1. population-oriented thinking, 2. caring neighborhood concept, and 3. smart-actions in co-creation [11]. The project plan provides a framework for the consortia by outlining the target population and the main six themes for action. The target population is divided into 1. the vulnerable multimorbid ill population, 2. people with one chronic disease and 3. people at risk for chronic disease and the healthy population. The six central themes for developing actions are 1. care programs, 2. care coordination, 3. medication, 4. caring neighborhoods, 5. assisted living and 6. health promotion [20]. The project plan therefore guides the local consortia in developing actions for integrated care however providing enough flexibility allowing stakeholders to experiment in finding the right local solutions.

### Selection of transitional care actions and interviewees

We selected actions with presumed impact on transitional care. Therefore, the researchers reviewed available information online (e.g. project plan) and developed a list of actions from ZZL that focused on improving transitional care pathways. This list was discussed with a project coordinator from ZZL and was subsequently narrowed down to four actions, which formed our final selection: 1. Care program heart failure to improve care for chronic heart failure patients by implementing four-guideline recommended interventions; 2. Intermediate care center to reduce the burden on hospital during the first wave of COVID-19; 3. Neighborhood teams to create close networks of local health care providers; and 4. Medication reconciliation envelope to provide a link between the hospital and the community pharmacy (See Table 1).

**Table 1:** Description of actions

Item List	Action 1: Intermediate Care Center	Action 2: Neighborhood teams	Action 3: Medication envelope action	Action 4: Care program chronic heart failure
**Objective**	To reduce the burden on hospitals during the first wave of COVID-19 and therefore to provide an immediate solution during the crisis	To create close networks of local health care professionals at neighborhood level in order to provide integrated care for patients and to detect vulnerable groups early on. The action aims to strengthen and structure primary care and improve cooperation between primary care, secondary care and tertiary care	To support the discharge process from the hospital by providing information on medication to the community pharmacist therefore allowing pharmacists to provide medication reconciliation to discharged patients	To improve care for heart failure patients by implementing four guideline-recommended disease management interventions
**Transitional care focus**	To provide support during two care transitions: 1) transition from hospital to intermediate care center and, 2) transition from intermediate care center to the community	To prevent unnecessary care transitions from the community setting to the hospital by connecting the neighborhood and providing early integrated care for vulnerable patients including coordination of care	To facilitate the information transfer on medications during the care transition from hospital to the community pharmacist	To avoid unnecessary care transitions to the hospital and improve necessary transitions from the hospital to the community
**Patient target group**	Vulnerable patients who were medically able to leave the hospital and who (often for social reasons) couldn’t go home yet - both patients with a COVID-19 infection or without an infection	Each neighborhood team decided on their own target population, however the focus was especially on the vulnerable or multimorbid population	All patients discharged from participating hospital departments	Heart failure patients or patients with a risk for heart failure living in the community or being admitted at the cardiology ward
**Main HCPs involved**	Coordinating pharmacist, community pharmacist, home care nurse, head nurse, social worker, GP, specialist, psychologist	Local health care professionals from the same neighborhood including: GP, pharmacist, physiotherapist, home care nurse, psychologist, tabaccologist, dietician, social worker	Nurse at discharge and community pharmacist	Heart failure educator, GP, heart failure nurse at the hospital ward, cardiologist, home pharmacist
**Key components**	• Coordination of medical, pharmaceutical and social care to arrange a seamless transition between the hospital and the intermediate care center and the intermediate care center and the home setting • The patients GP, pharmacist and home nurse were contacted and informed and if necessary a follow-up appointment was scheduled for the patient	Each neighborhood team developed specific aims and approaches for their patients that resulted in diverse projects such as: • group sessions to provide information on different topics (e.g. loneliness, healthy habits, positive health) • coaching sessions within the neighborhood such as walking moments or smoking cessation campaign for COPD patients • implementation of disease programs	• The patient receives an envelope from the nurse at discharge containing necessary documents to perform a medication reconciliation: medication scheme from the hospital, medication prescriptions at discharge and a registration form. The envelope is addressed to the community pharmacist. • To keep track of conducted medication reconciliations, community pharmacists are requested to scan the code of the envelope and to fill in a registration form	The following four interventions were implemented: 1. To improve the first-line diagnostics by reimbursing the natriuretic peptides test (NT-proBNP) for GP’s in Leuven which allows diagnosing heart failure 2. To implement automated diagnostic and qualitative audits for heart failure in primary care settings that help to safeguard high quality care for patients 3. To provide a heart failure education session that focus on self-care management for high-risk patients. GP’s or HCPs at the hospital can contact trained nurses to provide an education session to their patients 4. To improve the discharge moment for heart failure patients at the hospital by implementing a structured discharge protocol including: a checklist for high risk patients, telephone contact with the patient GP to plan follow-up appointment and heart failure education session 14 days after discharge
**Synergies**	• Siilo application: a secure medical messenger for HCPs • Medication envelope (Action 3)	• Siilo application • NexusHealthPro software: expending software access to several health care professional groups including physiotherapists, nurses, pharmacists, dentists and midwives. The software allows to consult hospital files of patients	/	• Medication envelope (Action 3)
**Implementation status**	The action was implemented during the first wave of COVID to provide an immediate solution during the crisis and was stopped afterwards	8 neighborhood teams are running in the region of ZZL and each team roughly covers a population of 5000–8000 inhabitants	The action is implemented within various departments of three hospital in the region/close to the region of ZZL	The action is implemented at various hospitals (cardiology department) and at the community (homecare and primary care)
**Context information**	Coordinating pharmacist organized the medication follow-up from admission until discharge. The role of the coordinating pharmacist was tested first in Belgium within the intermediate care center	The neighborhood teams were structured based on ‘natural’ networks in the community. A division of 24 small neighborhoods of 4000 inhabitants exist in Leuven and has been used to start the networking of neighborhoods within ZZL [21]	The action fills a current gap to digitally share information on medications at discharge	The four interventions have been tested previously in the Belgium setting [22]

For each action we first conducted an interview with the designated project coordinator from ZZL. Project coordinators were the first point of contact as they were involved in the development and implementation of each action and thus particularly knowledgeable. We also asked project coordinators to suggest relevant stakeholders from the field. We ensured to include stakeholders that were directly involved in the action. We continued to use the snowball method for additional selection of potential participants. Stakeholders were invited by e-mail including information about the study and an informed consent form. In total 24 invitations were send out of which 13 participants accepted the invitation.

### Data collection

Semi-structured interviews were carried out by two student researchers. The interviews were conducted in the timeframe from February until April 2021 via video conferencing software in order to oblige with the COVID-19 regulations. Two interview guides were developed: one for interviewing the project coordinators and one for interviewing other stakeholders. Questions were developed by reviewing relevant literature and in discussion with the research team. After each interview, the interview guide was revised and adjustments to the questions were made if deemed necessary. The main topics of the interview guide were: description of the action, role of the involved stakeholders, stakeholders perception on transitional care, sustainability of four actions, and lessons learned for the future of transitional care within ZZL. The complete interview guide can be found as Additional file 1. Also, we conducted a document analysis to collect additional data for the action description and searched for relevant documents using websites, actions plans, published papers and government reports. Included documents had to discuss at least one of the four selected actions.

Thirteen interviews were conducted in Dutch and one in English. The interviews lasted 55 minutes on average. The interviews were recorded and transcribed ad verbatim. English translations of transcripts were conducted by two student researchers and checked by a third researcher (ML). All transcripts were then imported to NVivo for data analysis.

### Data analysis

First, thematic analysis with an inductive approach following the six steps as described by Braun & Clarke 2006 was used to organize, describe and analyze the qualitative data in detail [23]. Second, collected data from the document analysis was added to the interview data in order to conduct the action description. This allowed for comparison of various sources in order to reach corroboration. Interview data of each action was analyzed separate for the action description. In order to analyze the experiences and perceptions of participants on transitional care, we analyzed interview data of all four actions combined. NVivo software was used to support the analysis process.

For the thematic analysis, first the familiarization of data was conducted by two researchers (ML, AF) reading and re-reading through the full transcripts to get a first understanding about the data as well as ideas for coding. Second, initial codes were generated from the data by one researcher (ML) and relevant data were collected for each code by coding all transcript interviews. A second researcher (AF) co-coded simultaneously all interviews independently. The researchers were meeting each other at three different time moments to discuss meaning of codes and resolve disagreements before, during and after coding. As a third step, themes were searched by listing all codes and starting the process to sort and combine codes leading to first ideas of themes and sub-themes. For the fourth step, themes were jointly reviewed by two researchers (ML, AF) to further refine themes and their meaning. A second revision with a third researcher (GG) was conducted and resulted in a thematic ‘map’. Defining, naming and describing themes separately and collectively was conducted in step five in discussion with three researchers (ML, AF, GG) and was discussed with the whole research team. Lastly, the final report was produced with supporting quotes from the data.

The Ethics Committee UZ Leuven/KU Leuven approved the study (registration number: MP017284) and the interviewees provided written informed consent.

## Results

### Interview participant characteristics

Fourteen interviews were conducted with thirteen stakeholders as one stakeholder was interviewed twice on two actions. Among the five project coordinators that were interviewed, there were three pharmacists, one physiotherapist and one general practitioner. The eight other stakeholders involved in the transitional care actions were two general practitioners, one home care nurse, one cardiac nurse, one cardiologist, one physiotherapist and one policy advisor for welfare & care. Additional file 2 presents an overview of interview candidates across the four actions.

### Transitional care actions

The thematic analysis and document analysis to describe four transitional care actions resulted in an item list that is presented for each of the four actions in Table 1. It includes the objective, transitional care focus, patient target group, main HCPs included, key components of the action, synergies, implementation status, context information. Additional file 3 lists all items and their meaning.

### Experiences and perceptions

Thematic analysis to explore the stakeholders experiences on transitional care resulted into the five themes: 1. Involvement of HCPs in decision making, 2, Improved community feeling – reduced barriers of communication, 3. Supporting tools and learning opportunities for HCPs, 4. Transitional care for patients in practice, 5. Key challenges: coordination, resources, financial & political support. Each theme is described separately in the next sections.

### Theme 1: involvement of HCP in decision making

HCPs appreciated being involved early on in shaping actions and welcomed the bottom-up approach from ZZL allowing them to propose own ideas for initiatives. Stakeholders felt motivated to be involved in all four actions that can benefit the care for their patients and bring themselves closer to local health care professionals from primary care and secondary care.

*“The approach that we are not going to come to you with a finished programme, but you are going to have a say in that programme. They really like that, they feel acknowledged…* “*(project coordinator).*

*“And a lot of specific questions, a lot of input from primary care providers. That was greatly appreciated, because it was actually the first time that the primary care providers had the feeling that they could directly take part in this process.” (project coordinator).*

Actions were considered as a potential leverage for change and positive experiences were shared with colleagues, who then convinced other HCPs to join**.** Moreover, HCPs appreciated that their input was taken into account while shaping actions and that for example a new role for coordinating pharmacists could be tested at the intermediate care center (Action 1). These positive changes triggered discussions on how health care professionals see their role and increased their confidence to be more assertive about being involved and voicing their opinion.

*“The neighborhood teams also have a very strong pull effect and that is because they are set up by the care providers themselves.” (project coordinator).*

*“They’re stronger, more assertive, and they now also asked to join the vaccination centers. That also makes communication more easy. They have an opinion […] “(Project coordinator).*

### Theme 2: improved community feeling - reduced barriers for communication

Across all actions, stakeholders reported that the threshold for communication had reduced and they noticed a significant improvement in the overall team spirit. HCPs experienced more direct and straightforward communication with each other.

*By doing so yes we have got to know each other, haven’t we? So now we know each other and yes we have a much lower threshold to send a Siilo message” (HCP 1).*

Also, HCPs perceived an increased openness for providing interdisciplinary care. Stakeholders reported that the actions change their way of thinking about interdisciplinary care and that patients with chronic care needs should not be treated alone.

*“There is much more openness to seeking and giving interdisciplinary advice “(HCP 1).*

*“And I think that this project has made me think more about the fact that chronic care, the people who need chronic care, should not be tackled alone. Um, that I should take steps towards other people and say, we’re going to tackle this more together.” (HCP 2).*

In addition, the actions allowed HCP to get to know each other and to build up trust over time within and between primary care and secondary care. This was often highlighted in Action 1 where care decisions were shared with all involved HCPs using the Siilo application. A feeling of professional joy and being proud of what was achieved together was reported by one stakeholder**.**

*“That professional joy that yes, everyone was really like that. I made my contribution here to a greater whole, even more than usual, you could really see that. Every little step that was taken by a particular care provider was shared in the group and everyone was like oh wow yes ok, we have to build on this. That was fantastic actually.” (HCP 3).*

### Theme 3: supporting tools and learning opportunities for HCPs

HCPs felt the necessity to have modern secure tools. The communication application Siilo was appreciated for small care teams allowing to solve misunderstandings, to make adjustments to the care plan or to receive information on medication in a quick and uncomplicated way.

*“I am convinced that Siilo can work on a small scale in defined patient groups” (HCP* 3).

Also, the paper envelope for conducting medication reconciliation was acknowledged by HCP as a tool for information transfer between hospital and the community pharmacy. Although many participants experienced these tools as useful, it was often stated that the current tools are seen as a temporary solution and a digital integrated patient record with messaging function is needed in the long-term.

*“The problem lies in the fact that we still have to work on paper. Both for the home care nurses and for the pharmacists as well. So in Belgium there is no safe, for the time being, no platform where we can work together in the same module to follow up on the treatment [...] ”(Project coordinator).*

HCP’s valued the learning opportunities that were provided within the actions. Stakeholders reported that the actions increased their awareness for the importance to conduct high-quality follow-up care (e.g. medication reconciliation guideline in action 3).

*“I actually think that this is an added value because the envelope campaign made us aware that we can get some extra information from the patient that we sometimes don’t think of ourselves” (HCP 4).*

### Theme 4: transitional care for patients in practice

Across all actions, stakeholders perceived that the actions offered support to guide their patients through complex treatments. Stakeholders experienced that the transitional care actions led to increased consultations with patients allowing HCPs for a better understanding of patient needs which ultimately resulted in delivering a more focused care approach.

*“But I think there is a lot more consultation and we can approach the care in a much more focused way and guide the people better” (HCP* 5).

*“Because you have a lot more, details about the person themselves. So I can really look more at what applies to this person. Not the generalized rattling off of questions to tell them. But with more information, you can indeed focus much more on the patient in front of you, in his individual context.” (HCP 4).*

In addition, stakeholders shared feedback from primary care professionals highlighting improved knowledge and awareness of patients in better understanding early signs and symptoms of their disease. This was observed for patients who received a heart failure session during follow-up (Action 4), leading to improved awareness and increased independence.

*“We have already heard from the home care nurses that the patients really do realize what they have to look out for. They are also becoming partly more independent, because they know that they have to start moving.” (HCP 5).*

Moreover, stakeholders highlighted that the actions within the neighborhood allowed patients to connect with their community by joining activities organized at the local level (e.g. Action 2 – walking/exercising moments, group sessions).

*“I think that at the moment, the impact of [name of neighborhood team]is certainly not that big on the things that you mention now. Erm, but it will rather have an impact on, erm, community work that brings people together more, such as the walks that are organized, the evenings that are organized around a particular theme” (HCP 6).*

Although the actions offered additional support for their patients, stakeholders overall felt that actions provided limited follow-up after transition and provided limited possibilities for patient involvement. Action 3 and 4 provided a one-time moment for follow-up which was perceived as too short by participants to ensure continuity of care.

*“But after that, it is up to the GP and the informal carer to follow up properly and there is no actual follow-up. There is no long-term follow-up within our project […]” (Project coordinator).*

Also, many stakeholders perceived patient involvement as difficult in practice, partly due to technological challenges and partly due to their own reluctance in being involved in their own care. Difficulties were mentioned in particular for older vulnerable patients as they were asked to login to a patient platform which was perhaps not the right solution for this population group and did not provide flexible solutions. Additionally, stakeholders observed that patients did not agree to be included in the actions likely due to limited reach and awareness of ZZL or the COVID-19 situation that led to many cancellations.

*“Um, but if we look at the chronic care population that I see, it’s mainly the 70-80 plus, yes. The whole internet thing, online login, all that stuff, is perhaps not so applicable to them. In 20 years’ time, it might be completely different, but at the moment it’s perhaps not perfectly adapted to the needs of the patient.” (HCP 2).*

*“It’s not always possible to really involve the patient according to the books, simply because sometimes there is a reluctance on the part of the patients and […]. I don’t mean to say that you can’t or you can’t do something about that, because that takes a lot of time, a lot of guidance” (HCP 7).*

### Theme 5: key challenges – communication, resources, financial & political support

A lack of concrete communication and coordination was reported, especially in the beginning of the pilot project as involved HCPs needed to take over new roles and responsibilities that often required to learn new skills. Therefore, bringing disciplines together and deciding on a common method for coordination was perceived as difficult.

*“because I had the impression that everyone was a bit on their own, all the disciplines were a bit on their own, but not enough working together” (Project coordinator).*

Additionally, stakeholders reported that a clear protocol for each action was missing, as a general project plan already existed that was used for all actions combined. This general project plan was set up in the development phase of the pilot project, however it was reported that the plan was modified in the meantime and not followed exactly as outlined. Stakeholders reported that the missing guidance created difficulties for implementation and decisions on ownership.

*“But because it didn’t have a very clear protocol of ‘yes, we’re going to do it like this and like that’. In practice, it was a bit difficult to decide who should take the initiative for which patients” (HCP 2).*

Moreover, the COVID-19 measurements impeded smooth communication and increased the effort needed to have regular meetings. For example, COVID-19 made it hard to onboard newly introduced roles such as coordinating pharmacists as all meetings were virtual and hence other HCPs were unaware of the existence of such roles.

*“So yes, that was what was difficult because of COVID, that not everyone’s role was equally clear […]” (Project coordinator).*

In addition to communication challenges, several structural challenges like increased workload of administrational tasks and shortness of staff was often mentioned. Also, due to the bottom-up approach from ZZL, HCPs active participation required extra energy and time, particularly in the early phase of the actions.

*“But then you also have the problem of resources. Neighborhood teams, that’s not really backed up by structural resources. The future of Zorgzaam Leuven itself is not yet very clear.” (policy maker).*

*“I think for some colleagues it’s an extra burden at the moment because it’s still under construction, so it takes energy to think about how we’re going to change that and what does that mean for my own practice” (HCP 1).*

Stakeholders also mentioned the lack of financial and political support. Financial resources were limited and there was limited regulatory leeway for projects to experiment with new care approaches. Moreover, stakeholders expressed that the lack of long-term perspective felt demotivating as no clear future and roadmap of ZZL was communicated making future planning of the various actions difficult.

*“Especially I think from the government and from, yes from government agencies more support I think or more also more shorter possibilities to tune things and roll them out. In such a way that, yes, we can also give a perspective to the people we are working with. I have the feeling that we are not able to offer enough perspective […]” (Project coordinator).*

## Discussion

In this qualitative case study we described four transitional care actions that are part of the pilot project ZZL and explored experiences and perceptions of involved stakeholders on transitional care in practice. Across the four actions, stakeholders valued active involvement and increased collaboration. Stakeholders brought forward that the co-designing approach of ZZL increased active participation as received input was taken into consideration when shaping actions. Additionally, actions encouraged interdisciplinary collaboration which in turn reduced barriers for communication and created a community feeling that benefitted professionals and patients. However, stakeholders emphasized key challenges, such as technological infrastructure, clear coordination, financial support and political support, that impeded sustainable implementation of actions. The analysis also showed that actions were often designed and implemented to provide (temporary) solutions to address these key challenges.

Our results highlight that HCPs intrinsic motivation and feeling connected with other HCPs facilitated the implementation of integrated care. In the literature this has been emphasized by the self-determination theory. The self-determination theory states that the satisfaction of the three basic psychological needs (autonomy, competence and relatedness) facilitates and sustains high quality motivation [24]. The need for autonomy refers to experience choice in the own role. Competence concerns the need to feel successful within the own role and to have the ability to develop necessary skills. The need of relatedness refers to the feeling of belonging and acceptance within a group [25]. Evidence has shown that applying the self-determination theory in the work context is linked to a wide range of positive outcomes for employees, such as developing autonomous work motivation [25]. Moreover, autonomous motivation is the most sustainable type of motivation, predicting high-quality performance and positive outcomes related to well-being [24, 26]. Our findings suggest that the pilot project ZZL created conditions that supported the three needs for self-determination. This was perceived as positive by HCPs and triggered their autonomous work motivation. In practice these needs were supported for involved HCPs namely by encouraging involvement in decision making, providing tools for communication or stimulating regular communication to build up trust.

The early involvement of stakeholders in the co-creation process encouraged stakeholders to share experience and increased collaboration which allowed to build up trust. Stakeholders had to get to know each other and learn new roles, such as designing and implementing actions. Therefore, the pilot phase of the project was used as a testing phase to experiment and to learn from mistakes. Actions that are undertaken in the pilot phase are first planned at small scale which is supposed to make the development and implementation of new actions less frightening and allows for correction of mistakes along the way [10]. The study of Fakha et al. (2022), a second study that analyzed the interview data on ZZL and focused on implementation research, supports the importance of involvement [27]. The study highlights engagement as the main implementation strategy used to back the implementation of the four transitional care actions. Also, motivated key individuals were identified as a crucial facilitating implementation factor. Using engaging activities allowed individuals to connect and build partnerships that strengthened motivation and commitment of HCPs, which was positively perceived by stakeholders as highlighted in our study. This is in line with further research stating that the approach to stimulate local dynamics of flexibility and experimentation is crucial, however that the successfulness depends on committed leaders and stakeholders [7].

While ZZL created several previously discussed motivating conditions for HCPs to bring change towards integrated care, various key challenges were identified that impeded further development, therefore leading to demotivation of HCPs. A key challenge that was identified in this study is the lack of integrated patient files in Belgium [26, 28]. Various digital systems were used by stakeholders and a lack of interoperability of these systems was stated, leading actions to develop temporary solutions to facilitate communication between HCPs. This finding is in line with other analyses on the Belgium health care context identifying the lack of shared electronic files as a major weakness hindering the development of integrated care in Belgium [13, 29]. Moreover, this resonates with findings of a recent systematic review indicating that none of the 15 reviewed transitional care models (TCMs) used electronic health records that allowed sharing of information between health care settings, stressing the need to create stronger digital links between settings [30]. Also the importance of using technologies within TCMs is underpinned by the SELFIE framework (Sustainable intEgrated chronic care modeLs for multi-morbidity: delivery, FInancing, and performancE), including shared information systems as a main component to facilitate care coordination of TCMs [31].

Furthermore, research shows that the lack of electronic health records tracking the patients decision-making process creates a barrier for patient engagement [29]. This is in line with our study findings as across all four actions patient engagement was described as limited. Stakeholders reported being reluctant towards patient engagement due to fear of increased workload and also described signs of reluctance of their patients. The findings are in line with a recent review highlighting the three main barriers for patient engagement as patient unwillingness, HCPs unwillingness and inadequate infrastructure [32]. The review concludes that attention should be paid to these barriers by creating a promoting environment ensuring sufficient resources and infrastructure and additionally establishing educational programs for patients and professionals [32].

### Study limitations

This has been the first study providing an initial understanding of what concrete actions have been locally implemented within one of the 12 Integreo projects in Belgium. These government initiated pilot projects are a huge undertaking, but have not been described in detail. To describe the four selected transitional care actions we used the two methods of document analysis and thematic analysis to reach corroboration and therefore increase trustworthiness.

The study findings also need to be interpreted in the light of some methodological considerations. In terms of the selection of stakeholders to be interviewed, only 13 of the potential 24 stakeholders agreed to participate in the study. They might have been more positive about the actions then those who were not interviewed. Also, the number of stakeholders interviewed per action varied with one action where we could only interview two stakeholders. We assume that this impact was limited as we did not analyze perceptions for each individual action separately, but made an overall analyses at the level of the four actions. Further, the total number of 14 interviews across 4 actions might lack representation, yet we collected meaningful data by interviewing project coordinators who are particular knowledgeable and who helped us to select key stakeholders that provided valuable insights. Lastly, we did not explore the views or experiences of patients involved in the transitional care actions and could therefore not critically discuss differences in experiences and perceptions between patients and HCPs. We strongly recommend to focus on the patient perspective in future research activities related to the evaluation of the Integreo projects. Moreover, we recommend a thorough evaluation of the effectiveness of ZZL and the other Integreo projects taking into account the quadruple aim objectives, which was defined as the ultimate goal of Integreo. Additionally, we suggest to continue to conduct process evaluations to better understand how the outcomes were achieved in order to build on lessons learned and adapt actions. We suggest to regular evaluate the development of pilot projects as they are dynamic, change over time and are context-specific.

## Conclusion

To conclude, our findings indicate that the integrated care project ZZL created conditions to promote autonomous work motivation for HCPs. The project encouraged the development of multidisciplinary care networks at the local level, which allowed professionals to connect and create partnerships. Also, the project offered (temporary) solutions to address pressing problems for continuity of care. Yet, key challenges were identified that impeded long-term planning for integrated care within the project region. Additionally, our analysis highlights how integrated care and transitional care are interconnected, by describing how the four actions linked to the integrated care project addressed various transitional care components to achieve continuity of care for patients.

## Supplementary Information

**Additional file 1.** Semi-structured Interview guide.

**Additional file 2: Table.** Characteristics of interviewees.

**Additional file 3: Table.** Action description item list.

## Data Availability

The anonymised data are available from the corresponding author upon reasonable request.

## Abbreviations

HCP(s): Health care professional(s)
Integreo: Integrated Care for Better Health
ZZL: Zorgzaam Leuven
TCM(s): Transitional care model(s)
SELFIE: Sustainable intEgrated chronic care modeLs for multi-morbidity: delivery, FInancing, and performancE
