# Supplementary material for: Stakeholders’ experiences and perception on transitional care initiatives within an integrated care project in Belgium: a qualitative interview study
Source: BMC Geriatr. 2023 Jan 23;23:41. doi: 10.1186/s12877-023-03746-z (PMC9868499; doi:10.1186/s12877-023-03746-z)
Supplement: Supplementary file 1 — Additional file 1. Semi-structured Interview guide. [file 12877_2023_3746_MOESM1_ESM.pdf]

Additional File 1.

Semi-structured Interview guide

(Note – the underlining questions are the ones to consider for this study as they refer to describe actions and explore experiences and perceptions on transitional care. The other questions were used for a second study investigating the implementation of the four actions [27])

Questions – Project coordinator

1. Can you shortly describe the action and your role in those actions?
2. Which stakeholders and healthcare professionals were involved in those actions?
3. What did u do to encourage, motivate and engage healthcare professionals to commit to using these actions? Did you assign any champions, leaders, persons with a facilitator role, or any other assigned roles to help spread those actions to the healthcare providers?
4. Now we will focus on the aspect transition of care for elderly. What does transitional care mean for you?
5. Can you shortly describe the impact of the action on the transition of care for elderly people? (e.g. communication, shared-decision making, patient involvement, informal caregiver involvement, person-centered care, medication reconciliation, continuity of care at home – organization of follow-up care)
6. Can you shortly describe the impact of the action on the healthcare professionals?
7. Can you shortly describe the impact of the action on the healthcare system?
8. To what extent are those actions fully integrated? What stage are they at?
    a. Status of implementation
    b. Does the current state of implementation of those actions meet your expectations?
    c. Is the action still carried out as planned?
        i. How has the action been affected in the context of COVID-19
        ii. Sustainability of the project
9. Did u develop a project plan to implement and roll out those actions to the various healthcare providers? If yes, can you briefly describe the plan?
    a. Did you perform any activities or use specific strategies to implement those actions? (e.g.: education and training, champions, mandate change)
10. Did you already receive some feedback from primary and secondary healthcare providers? If yes, what did you learn about it?
11. Adoption (= intention to try to use the intervention)

   a. How would you assess (or what would you say) on the adoption level of these actions by healthcare professionals? This means the intention to use the intervention by the healthcare professionals.
   b. What are the barriers/obstacles/challenges in the implementation of those actions? And were there any facilitators to enable a better and successful implementation of those actions?

12. What is needed to continue these actions?
13. How can this action be expanded?
14. Which lessons could be learned from this action regarding the future?

Completing the interview

Would you like to add something to this interview?
Thank you for your participation.

Questions – Healthcare professional

1. Can you shortly describe the action and your role in those action?
   a. How do you experience this role?
2. Which other persons are involved?
   a. How did you experience the collaboration and the communication? Is everyone aware of his/her responsibilities?
3. Now we will focus on the aspect transition of care for elderly. What does transitional care mean for you?
4. Can you shortly describe the impact of the action on the transition of care for elderly people? (e.g. communication, shared-decision making, patient involvement, informal caregiver involvement, person-centered care, medication reconciliation, continuity of care at home – organization of follow-up care)
5. Can you shortly describe the impact of the action on the healthcare professionals?
6. Can you shortly describe the impact of the action on the healthcare system?
7. Acceptability (perceived views that an intervention is agreeable, satisfactory, credible, and comfortable): to what extent do you think those actions are satisfactory and advantageous to the older persons with chronic disease and requiring care transitions between hospital & home?
8. Appropriateness (perceived compatibility of the intervention with needs & practices of a setting or population): to what extent do you think these actions address/meet the care needs of older persons with chronic disease and requiring care transitions?
9. Is the action still performed as planned?

    a. Status of implementation
    b. How has the action been affected in the context of COVID-19?
    c. Sustainability of the action
10. Experiences and expectations:
    a. How did you experience those actions?
    b. Is the action meeting your expectation? Can you explain the reasons as to why it is or isn't meeting your expectations?
    c. According to you, do you think the implementation of the action is successful? Can you explain the reasons as to why the implementation is or isn't successful?
11. What are the barriers/obstacles/challenges in the implementation of those actions? And were there any facilitators to enable a better and successful implementation of those actions?
12. Which lessons could be learned from this action regarding the future?

Completing the interview

Would you like to add something to this interview?
Thank you for your participation.
