# Supplementary material for: Stakeholders’ experiences and perception on transitional care initiatives within an integrated care project in Belgium: a qualitative interview study
Source: BMC Geriatr. 2023 Jan 23;23:41. doi: 10.1186/s12877-023-03746-z (PMC9868499; doi:10.1186/s12877-023-03746-z)
Supplement: Supplementary file 2 — Additional file 2: Table. Characteristics of interviewees. [file 12877_2023_3746_MOESM2_ESM.pdf]

Additional File 2.

Table: Characteristics of interviewees

Number of interviewees	Coordinator or stakeholder	Profession of interviewees	Action number
1	Coordinator	Pharmacist	1
2	Coordinator	Pharmacist	1
3	Coordinator	Pharmacist	1
4	Stakeholder	Home nurse	1
5	Coordinator	Physiotherapist	2
6	Stakeholder	GP	2
7	Stakeholder	GP	2
8	Stakeholder	Physiotherapist	2
9	Stakeholder	Policy advisor	2
2	Coordinator	Pharmacist	3
10	Stakeholder	Pharmacist	3
11	Coordinator	GP	4
12	Stakeholder	Cardiologist	4
13	Stakeholder	Nurse	4
