# Supplementary material for: Stakeholders’ experiences and perception on transitional care initiatives within an integrated care project in Belgium: a qualitative interview study
Source: BMC Geriatr. 2023 Jan 23;23:41. doi: 10.1186/s12877-023-03746-z (PMC9868499; doi:10.1186/s12877-023-03746-z)
Supplement: Supplementary file 3 — Additional file 3: Table. Action description item list. [file 12877_2023_3746_MOESM3_ESM.pdf]

Additional File 3.

Table: Action description item list

Item description	
1. Objective	Action purpose
2. Transitional care focus	How actions improve transitional care for patients and across what care settings
3. Patient target group	Patient groups that are targeted with the actions
4. Main HCPs involved	List of all HCPs involved in delivering the actions
5. Key components	Essential pillars that define the actions
6. Synergies	Possible links with other actions or with existing care
7. Implementation status	Implementation status at the moment of data collection: actions ongoing or stopped and locations of implementation
8. Context information	Unique local aspects that need to be known to understand the actions
